# Response to the Letter to Editor: “Do Inter-Country Differences in the Frequency of Abusive Head Trauma Reflect Different Proportions of Overdiagnosis of Abuse or True Differences in Abuse?”

**DOI:** 10.2188/jea.JE20190106

**Published:** 2020-06-05

**Authors:** Yui Yamaoka, Takeo Fujiwara, Yoshihisa Fujino, Shinya Matsuda, Kiyohide Fushimi

**Affiliations:** 1 Center on Child Abuse and Neglect, University of Oklahoma Health Sciences Center, Oklahoma, USA; 2 Department of Global Health Promotion, Tokyo Medical and Dental University, Tokyo, Japan; 3 Department of Environmental Epidemiology, Institute of Industrial Ecological Sciences, University of Occupational and Environmental Health, Kitakyushu, Japan; 4 Department of Preventive Medicine and Community Health, School of Medicine, University of Occupational and Environmental Health, Kitakyushu, Japan; 5 Data Science Center of Occupational Health, University of Occupational and Environmental Health, Kitakyushu, Japan; 6 Department of Health Policy and Informatics, Tokyo Medical and Dental University, Tokyo, Japan

We appreciate the editors for the opportunity to respond to the letter by Dr. Ulf et al^1^ regarding our article.^2^ Their concerns focused on two areas: 1) the possibility of overdiagnosis of abusive head trauma (AHT) by underestimating the presence of Benign External Hydrocephalus (BEH) and 2) the validity of International Classification of Disease (ICD) codes developed by the United States Centers for Disease Control (CDC) in defining AHT.

BEH, or so-called benign enlargement of subarachnoid space (BESS), is commonly seen on imaging studies for macrocephaly of young children. It is a transient developmental phenomenon characterized by enlargement of the extra-axial fluid spaces, which spontaneously resolves by 2 years of age.^3^ The incidence rate of BEH can be estimated about 0.4 per 1,000 live births in population-based study.^4^ Clinicians and researchers have postulated a relationship between BEH/BESS and subdural hemorrhage (SDH) or subdural collections (SDC), with minimal or no trauma.^5^^,^^6^ SDC is used as a nonspecific umbrella term for findings within the subdural space, including SDH, subdural hygroma (SDHy), subdural hematohygroma (SDHHys), chronic subdural hematoma (cSDH), subdural effusion, and empyema.^7^ Recent studies reported infrequent presence of SDH/SDC among children with BEH/BESS; that is, SDC was seen among 5.6% of 108 children with BESS^8^ and 5.8% of 311 imaging studies of children with BESS.^9^ As Ulf et al mentioned, we cannot deny the possibility of over-diagnosing possible AHT due to limitation of using available ICD-10 codes from DPC database. Considering the incidence rate of BEH/BESS,^4^ there might be approximately 400 children with BEH/BESS born each year because the annual number of live births is about one million in Japan. Using a 6% prevalence of SDC among children with BEH/BESS, 24 of them might develop SDC annually in Japan. If we would exclude these estimated 96 children with BEH/BESS and SDH from our 4-years samples (ie, estimating 1,329 possible AHT), the incidence of possible AHT can be calculated as 38.94 (95% CI, 38.88–39.00), which is similar to our original findings.

One recent study reported that more than one-third of children with BESS and SDH-mild symptoms had concomitant suspicious injuries (ie, retinal hemorrhage, bruising, fracture, or abdominal injury).^6^ BEH/BESS with SDH/SCD cannot always exclude the possibility of abuse, and we should consider to workup for the evaluation of non-accidental trauma. Therefore, we concluded that the incidence rates, 41.7/100,000 for possible AHT and 7.2/100,000 for presumptive AHT, were “at most” estimations using available dataset in the current Japanese situation.

They also mentioned the concern about two peaks of month-age distribution. The second peak at 8 months old would be a similar peak age for BEH/BESS,^4^ but still presumptive AHT, having SDH and physical abuse/retinal hemorrhage, had the same second peak. Sudden Infant Death Syndrome (SIDS) is not relevant to this discussion because SIDS is diagnosed with absence of clinical and pathological findings via autopsy (ie, absence of SDH). Further studies that include active surveillance are needed to reveal more accurate incidence and to explore mechanisms for the occurrence of AHT in later infancy.

In terms of ICD-9-CM/ ICD-10 codes in defining AHT, CDC developed the definitions with a broad panel of professionals, including pediatricians, child maltreatment experts, abusive head trauma experts, injury surveillance experts, ICD coding experts, and experienced state health department personnel.^10^ Berger et al^11^ examined 223 children to assess the accuracy of ICD-based operational definition compared to AHT evaluated by a hospital-based Child Protection Team. The sensitivity and specificity of ICD codes were 92% and 96%, respectively.^11^ Parrish et al^12^ also reported 91% of sensitivity and 99% of specificity for using ICD codes based on CDC definition when they applied it to the multi-source passive surveillance system and compared with medical chart reviews. Although active surveillance (ie, direct case ascertainment with clinical assessment) is a common golden standard to capture and evaluate AHT, it usually requires greater amounts of time and funding on a large scale. The high sensitivity and specificity of using ICD codes suggests its usefulness for the research purpose, especially to evaluate prevention programs, in population-based study.

In terms of the systematic review of AHT by the Swedish Agency of Health Technology Assessment and Assessment of Social Services (SBU),^13^^,^^14^ we consider it is a different matter from using ICD-10 codes as a passive surveillance in the current epidemiological study. SBU’s report insisted that there is insufficient scientific evidence for using “triad: subdual hematoma, retinal hemorrhage, encephalopathy” for diagnosing AHT in the medicolegal system. However, the reliability of this systematic review itself has been questioned in terms of improper systematic review questions, improper criteria for assessing bias, and inequitable application of quality of study assessment standards.^15^ After various clinicians and researchers similarly expressed concerns toward it,^16^^–^^20^ a consensus statement on AHT was published and endorsed by a broad range of international academic societies.^21^ It mentioned that the diagnosis of AHT is a complex process requiring historical, clinical, laboratory, and radiological examinations by a multidisciplinary team. These careful considerations are important for direct case ascertainment in clinical setting or utilizing actual cases for an active surveillance, but do not fit in discussions of our current research methodology. The epidemiological surveillance has different perspectives in defining the AHT cases from medically diagnosing AHT or legal decision-making processes. Even though our inclusion criteria for presumptive/possible AHT has limitations, as we mentioned in discussion, we reported the current incidence of hospitalized AHT using ICD codes based on CDC definitions from reasonably available claims dataset as a population-based sample. Further research is still needed to capture more epidemiologically-accurate incidence of AHT by improving data quality and quantity in Japan.
